# Toward a Predictive Model of Success in Contingency Management: A Proof of Concept Study Utilizing Behavioral Economic, Clinical Severity, and Alcohol Use Severity Measures

**DOI:** 10.1007/s40732-025-00671-y

**Published:** 2026-02-08

**Authors:** Haily K. Traxler, Christopher T. Franck, Mikhail N. Koffarnus

**Affiliations:** 1 Department of Behavioral Science, University of Kentucky, 845 Angliana Avenue, Lexington, KY 40508 USA; 2 Department of Statistics, Virginia Tech, Blacksburg, VA USA; 3 Department of Family and Community Medicine, University of Kentucky, 2195 Harrodsburg Rd, Suite 125, Lexington, KY 40504 USA

**Keywords:** Contingency management, Behavioral economics, Alcohol use disorder, Substance use disorders

## Abstract

As contingency management (CM) moves from research to practice, researchers have a responsibility to outline the minimum procedural necessities that lead to an effective, sustainable treatment that can be implemented as a mainstream therapy for substance use disorders. To begin identifying the minimum requirements, the purpose of the current study was to provide framework and a first step toward building a risk calculator that predicts treatment outcomes in CM, and can predict the optimal incentive size to prescribe by evaluating behavioral economic factors, demographic variables, and use severity measures in individuals who completed CM treatment for alcohol use. Participants were 38 individuals enrolled in the active treatment arms of two parent CM studies for reducing alcohol use (Koffarnus et al., 2018; Koffarnus et al., 2021). Participants were 42 years old on average, 55% male, and a majority were white, non-Hispanic. Fifteen candidate predictor variables were assessed for inclusion in the predictive model including demographic variables, use severity scores, and behavioral economic parameters. A logistic regression framework was used to identify top predictive models. Accuracy was assessed by computing receiver operating characteristic (ROC) curves and area under the curves. A model including the delay discounting parameter, log_10_(ED50) of alcohol, participant age, and Beck’s Anxiety Inventory score was predictive of treatment outcomes in the current sample. The results demonstrate the utility of the ROC analysis as a method for identifying a predictive model. Further research is needed to replicate and verify the findings of the current analysis.

## Introduction

Over the past several decades, a growing body of research has demonstrated the robust effectiveness of contingency management (CM) as an intervention to treat substance use disorders (SUD; De Crescenzo et al., 2018; Dutra et al., 2008; Ginley et al., 2021). In 2021, the Biden-Harris Administration announced seven drug policy priority areas, including the expansion of evidence-based interventions like CM (White House, 2021). Since the announcement, several states, including California, Washington, Montana, Hawaii, and Delaware have begun test-running CM using Medicaid as a payor, with other states (i.e., Michigan, Rhode Island) awaiting approval to begin piloting (see Kaufman et al., 2025). Private companies (e.g., DynamiCare Health) have also begun using CM to treat a range of SUD. As CM continues to move from research to practice, researchers have an important responsibility to outline the minimum procedural necessities that will lead to an effective, sustainable treatment that can be implemented as a mainstream therapy for SUD. CM is a highly manipulable intervention. Modifiable components of CM include the incentive size and type, schedule of reinforcer delivery, targeted behaviors and their measurement, and treatment modality (e.g., virtual vs. in-person, prize bowl vs. vouchers). In effort to identify the minimum requirements of a widely applicable CM intervention, the purpose of the current study was to initiate a first step toward building a risk calculator that predicts treatment outcomes in CM for alcohol use disorder (AUD) based on a range of participant factors (e.g., demographics, substance use characteristics, assessment scores), and can therefore predict the optimal incentive size to prescribe to increase the probability of success in CM.

The extant literature base for CM highlights its potential as a first-line treatment for SUD. Prior research shows that remotely delivered CM effectively reduces alcohol use (Coughlin et al., 2023). Furthermore, CM for alcohol use has been specifically shown to improve treatment adherence in people with AUD (Biswal et al., 2024). However, all substance use treatments are susceptible to pitfalls such as treatment nonresponsiveness by some consumers. Thus, it is important to identify the factors associated with treatment response. Identifying these factors may help providers to tailor CM interventions, ensuring a greater success rate for any given individual. Identifying these factors can bring awareness to these variables and lead to special focus on tailoring interventions accordingly. Foster et al. (2019) reviewed existing literature to assess the individual factors that were associated with treatment response in people who used stimulants or opioids. They identified several factors associated with positive, mixed, and negative treatment responses in CM, as well as factors that had no impact on treatment response. However, these results were largely descriptive and more work is needed to identify the optimal conditions under which CM is effective for a range of substances. Among factors that may be especially important for identifying responsiveness to treatment are behavioral economic variables.

Reinforcer pathologies are defined as the joint effects of high valuation and preference for immediate consequences even when long-term consequences are negative (Bickel et al., 2014). Behavioral economics provides a framework for studying reinforcer pathologies of substance use. Behavioral economic procedures (i.e., delay discounting, demand) quantify decision making in relation to delay and cost. Delay discounting measures choice between smaller, immediate outcomes versus delayed, larger outcomes. Steeper delay discounting (i.e., greater preference for smaller, immediate consequences) is associated with excessive substance use (see MacKillop et al., 2011). Mazur’s (1987) delay discounting function

1 $$V= \frac{A}{1+kD}$$

describes the reinforcer value, *V*, wherein *A* is the amount of the reinforcer, *D* is the delay to reinforcement, and *k* is the discount rate. The 5-Trial Adjusting Delay Task was used in the current study to identify the indifference point between preference for the smaller, sooner and larger, delayed rewards (see Koffarnus & Bickel, 2014). The 5-Trial Adjusting Delay Task takes less than 1 min on average to complete. It directly measures the ED50 value, which is the delay that effectively reduces the value of the reinforcer by 50% (Yoon & Higgins, 2008). This midpoint value is less susceptible to floor and ceiling effects than other discounting parameters. It is the inverse of the discounting rate *k* (Yoon & Higgins, 2008). The 5-Trial Adjusting Delay Task differs from other discounting tasks in that only the delay is manipulated, whereas the reward magnitudes are held constant. This allows for a straightforward analysis of both monetary and nonmonetary discounting, because nonmonetary rewards are presented as logical amounts (e.g., 1 serving of alcohol), rather than fractions of the reward (e.g., 0.13 serving of alcohol; Koffarnus & Bickel, 2014).

Demand analysis is a second tool used to study reinforcer pathologies. Demand analysis characterizes consumption as a function of cost. Cost is systematically increased through manipulating the monetary cost or response requirement for obtaining the commodity of interest. Demand tasks are implemented in a variety of formats, such as human operant tasks that require individuals to engage in increasing Fixed Ratio requirements to obtain a reward (e.g., Rowlett, 2000). Demand tasks can also be implemented in survey form through hypothetical or real purchase tasks (e.g., Zvorsky et al., 2019). For example, a participant completing a hypothetical purchase task may be asked to indicate the number of hypothetical alcoholic drinks they would purchase across an array of prices ranging from $0 (free) to $100 per drink. The exponentiated model of demand,

$$Q={Q}_{0}*{10}^{s{(e}^{-\alpha {Q}_{0}C}-1)}$$

describes consumption (*Q*), where *Q*_*0*_ is consumption as price approaches zero, *α* is the rate of change in elasticity, and *C* is the price of the commodity. The scaling constant *s* specifies the logarithmic range of the data^1^ (Koffarnus et al., 2015).

Prior research suggests that behavioral economic procedures may be useful in predicting treatment initiation and perceived likelihood of achieving abstinence in CM using a sample of participants on Amazon Mechanical Turk (Traxler et al., 2023). Prior studies have shown that higher alcohol demand is associated with greater severity of AUD and poor response to treatment (e.g., Bickel et al., 2014; Tucker et al., 2016). In addition, steeper discounting is associated with AUD (e.g., Minhas et al., 2020; Petry, 2001). The purpose of the current proof-of-concept study was to provide framework and first step toward building a risk calculator that predicts treatment outcomes in CM by evaluating behavioral economic factors, demographic variables, and clinical severity measures in individuals who completed CM treatment for alcohol use. By including all of these variables, we may be able to identify specific participant profiles that may require more support to achieve success in treatment. The current analysis utilizes a small sample size of participants from previously published CM experiments to pilot the analytic procedures.

## Methods

### Participants

Data from 38 participants from two parent pilot studies were included in this analysis. Twenty participants received remotely delivered incentive-based CM for alcohol use (for full methods, see Koffarnus et al., 2018). Eighteen participants received CM for alcohol use using deposit contracts (for full methods, see Koffarnus et al., 2021). In both studies, participants were included if they were at least 18 years old, met *DSM-V* criteria for AUD, and expressed desire to cut down or quit drinking. Participants were excluded if they scored greater than 23 on the Alcohol Withdrawal Symptoms Checklist (AWSC). In addition, participants enrolled in Koffarnus et al.,’s 2018 study were excluded if they met *DSM-V* criteria for substances other than alcohol, caffeine, or nicotine. Participants enrolled in Koffarnus et al.,’s 2021 study were included if they reported willingness to deposit $75 into an incentive fund and excluded if they met criteria for substances other than alcohol, caffeine, marijuana, or nicotine. Table 1 displays overall and study specific inclusion/exclusion criteria. We report how we determined our sample size, all data exclusions, all manipulations, and all measures in the study.

**Table 1:** Inclusion and exclusion criteria overall and study specific

	Overall (*N* = 38)	Contingent Incentives* (*N* = 20)	Deposit Contracts** (*N* = 18)
Inclusion	• At least 18 years old • *DSM-V* criteria for AUD • Desire to cut down or quit drinking alcohol		• Willing to provide $75 monetary deposit in an incentive fund
Exclusion	• AWSC score > 23	• *DSM-V* criteria for substances other than alcohol, caffeine, nicotine	• *DSM-V* criteria for substances other than alcohol, caffeine, marijuana, nicotine

Inclusion and exclusion criteria in the overall column are applied to both Contingent Incentives and Deposit Contracts groups. AUD = Alcohol Use Disorder; AWSC = Alcohol Withdrawal Symptom Scale; *data from Koffarnus et al., 2018; **data from Koffarnus et al., 2021

### Materials

Data were analyzed using R software version 4.3.1. The *tidyverse* (Wickham, 2023), *bestglm* (McLeod et al., 2022), *pROC* (Robin et al., 2023), and *ROCR* (Sing et al., 2020) packages were used to develop and cross-validate the predictive model.

### Procedures

Participants were individuals randomized into active CM conditions in each of the parent studies. Each parent study included a control group, but control group data were excluded from the analysis because they did not provide predictive information about CM outcomes. In addition to the CM intervention, participants in both studies completed a battery of assessments to characterize demographic variables and substance use behaviors. The results of these studies were combined for the current analysis to produce a predictive model of treatment outcomes.

### Data Analysis

For the current analysis, the proportion of days in which breath alcohol levels (BAL) measured less than 0.02 for alcohol was calculated. Treatment success, the outcome variable in this study, was defined as 80% or more breath samples submitted with BAL < 0.02. This success criterion was selected to capture the behavior of participants who met the contingency a majority of the time, allowing for some flexibility and reflecting a model of harm reduction (i.e., reduced use) rather than complete abstinence. This is a binary variable, with 1 indicating success and 0 indicating nonresponsiveness to CM. In total, 73.7% of participants achieved success in CM.

Fifteen candidate predictor variables were assessed for inclusion in the predictive model. These factors included demographic variables, scores on various assessments, and behavioral economic parameters of delay discounting and demand. A binary variable for CM group was also included (1 = contingency management, 2 = deposit contracts). Demographic factors included gender, age, ethnicity, education, and income. The square root of income was used to tighten the distribution of income and reduce the influence of outliers.

Assessment scores from the Obsessive–Compulsive Drinking Scale (OCDS), the Alcohol Use Disorder Identification Test (AUDIT), the Alcohol Withdrawal Symptoms Scale (AWSC), Beck’s Anxiety Inventory (BAI), and Beck’s Depression Inventory (BDI) were included. The OCDS is a 14-item assessment that measures obsessive thoughts about and compulsive behaviors toward alcohol use, with greater scores indicating greater symptom severity (Anton et al., 1995). The AUDIT is a 10-item assessment used to measure alcohol use severity, with scores 0–7 indicating low risk alcohol use, 8–15 indicating medium risk, 16–19 high risk, and 20 + indicating that addiction is likely (Babor et al., 2001). The AWSC is a 17-item self-rated instrument used to quantify alcohol withdrawal symptoms within the last 24 h. Symptoms are rated on a scale of 0–4. Sum scores greater than 23 correspond to threshold for urgent anti-withdrawal medication (Pittman et al., 2007). The BAI is a 21 item self-report measure of anxiety, with scores 0–7 indicating minimal anxiety, 8–15 mild anxiety, 16–25 indicating moderate anxiety, and scores 25 or higher indicating severe anxiety (Beck et al., 1988). Finally, the BDI is a 21-item self-report measure of depression, with scores 0–9 indicating minimal depression, 10–18 indicating mild depression, 19–29 indicating moderate depression, and scores 30–63 indicating severe depression (Beck et al., 1961).

Delay discounting variables included the log_10_(ED50) for alcohol discounting and the log_10_(ED50) for monetary discounting of $1,000. Finally, demand variables included log_10_(Q_0_) and log_10_(*α*). Behavioral economic parameters were estimated in log-space to minimize issues of convergence (see Rzeszutek et al., 2022; Traxler et al., 2023).

To develop a predictive model of treatment success, the *bestglm* package was used to identify the top predictive models in a logistic regression framework. Model selection was based on Bayesian Inference Criterion (BIC). Posterior model probability (i.e., the probability that each model is the correct one) was computed using the approximation based on BIC described here (Kass & Raftery, 1995). We used a uniform prior on the model space. The top five models were reported. Accuracy was assessed by computing receiver operating characteristic (ROC) curves and computing the area underneath those curves (AUC). Results were verified using leave-one-out cross-validation. Data and *R* code are available at 10.17605/OSF.IO/U8GKQ.

## Results

### Demographics

Table 2 displays overall and study-specific demographic data. On average, participants were 42 (SD = 12.4) years old. The average education was 14.3 years (*SD* = 2.3). About 55% of participants were male. A majority were white and non-Hispanic. Median monthly income was $1,900 (*SD* = $2,369.16). Participants in Koffarnus et al.,’s 2018 study were significantly older (46.6 years old) than those in the 2021 study (36.1 years old; *p* <.01). Other study-specific demographic variables were nonsignificant.

**Table 2:** Demographic data for participants from contingent incentives and deposit contracts groups

Factor	Overall	Contingent Incentives	Deposit Contracts
Age (*M* [*SD*])	41.6 (12.4)	46.6 (12.5)	36.1 (9.9)**
Education (years) (*M* [*SD*])	14.3 (2.3)	14.5 (2.5)	14.1 (2.1)
Gender (*N* [%])			
Male	21 (55%)	13 (65%)	8 (44%)
Female	17 (44.7%)	7 (35%)	10 (55.6%)
Race (*N* [%])			
White	30 (78.9%)	16 (80%)	14 (77.8%)
African American	5 (13.2%)	4 (20%)	1 (5.6%)
Other	3 (7.9%)	0	3 (16.7%)
Ethnicity (*N* [%])			
Hispanic	1 (2.6%)	0	1 (5.6%)
Non-Hispanic	37 (97.4%)	20 (100%)	17 (94.4%)
Monthly Income (*Median* [*IQR*])	1900 [769.3–3525]	2750 [787.50–11200]	1550 [819.3–4000]

*IQR* Inter-quartile range; **Contingent incentives and deposit contracts groups significantly differed in age, *p* <.01

### Model Selection Analysis

Prior to running the model selection analysis, scatterplots for all factor pairings were examined to assess multicollinearity. Pearson’s correlation coefficients were below.8 in magnitude for all pairings. The set of models under consideration consisted of 15 candidate predictors. Because each variable is either included or not included in a model, there are 2^15^ – 1 = 32,767 possible models. One is subtracted from the total because we do not consider the intercept-only model. The top model as determined by BIC included BAI score, age, and log_10_(ED50) for alcohol discounting as predictors, with a BIC of 35.2. The BIC for the next best fitting model was 35.7. Posterior probabilities were calculated to analyze differences between the top two models. The posterior probability for the top model was 0.039 and 0.031 for the second model. BIC and posterior probability for the third best model were 36.8 and 0.017, respectively. Table 3 displays BIC and posterior probability for each of the five top models identified.

**Table 3:** Top five predictive models

Model	Factors	BIC	Posterior Probability
1	BAI score, Alcohol log_10_(ED50), Age	35.2	0.039
2	BAI score, Alcohol log_10_(ED50)	35.7	0.031
3	BAI score, Alcohol log_10_(ED50), log_10_*Q*_*0*_	36.8	0.017
4	BAI score, Alcohol log_10_(ED50), log_10_(*α*)	37	0.016
5	BAI Score, Alcohol log_10_(ED50), AWSC score	37.1	0.015

Participants who met treatment success criteria had a BAI score of 9.1 (*SD* = 7.1), were 42.3 (*SD* = 11.7) years old, and a log_10_(ED50) value of 1.6 on average. Participants who did not meet treatment success criteria had a BAI score of 4.1 (*SD* = 5.93), were 39.6 (*SD* = 14.7) years old, and had a log_10_(ED50) value of 0.47 on average. BAI scores for the successful group fell within the mild range, whereas scores for participants who did not meet treatment success fell in the “low anxiety” range.

**Fig. 1:**
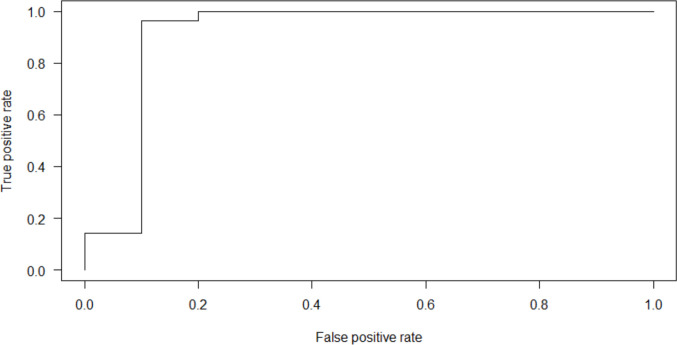
Receiver operator curve displaying false positive rate by true positive rate

**Fig. 2:**
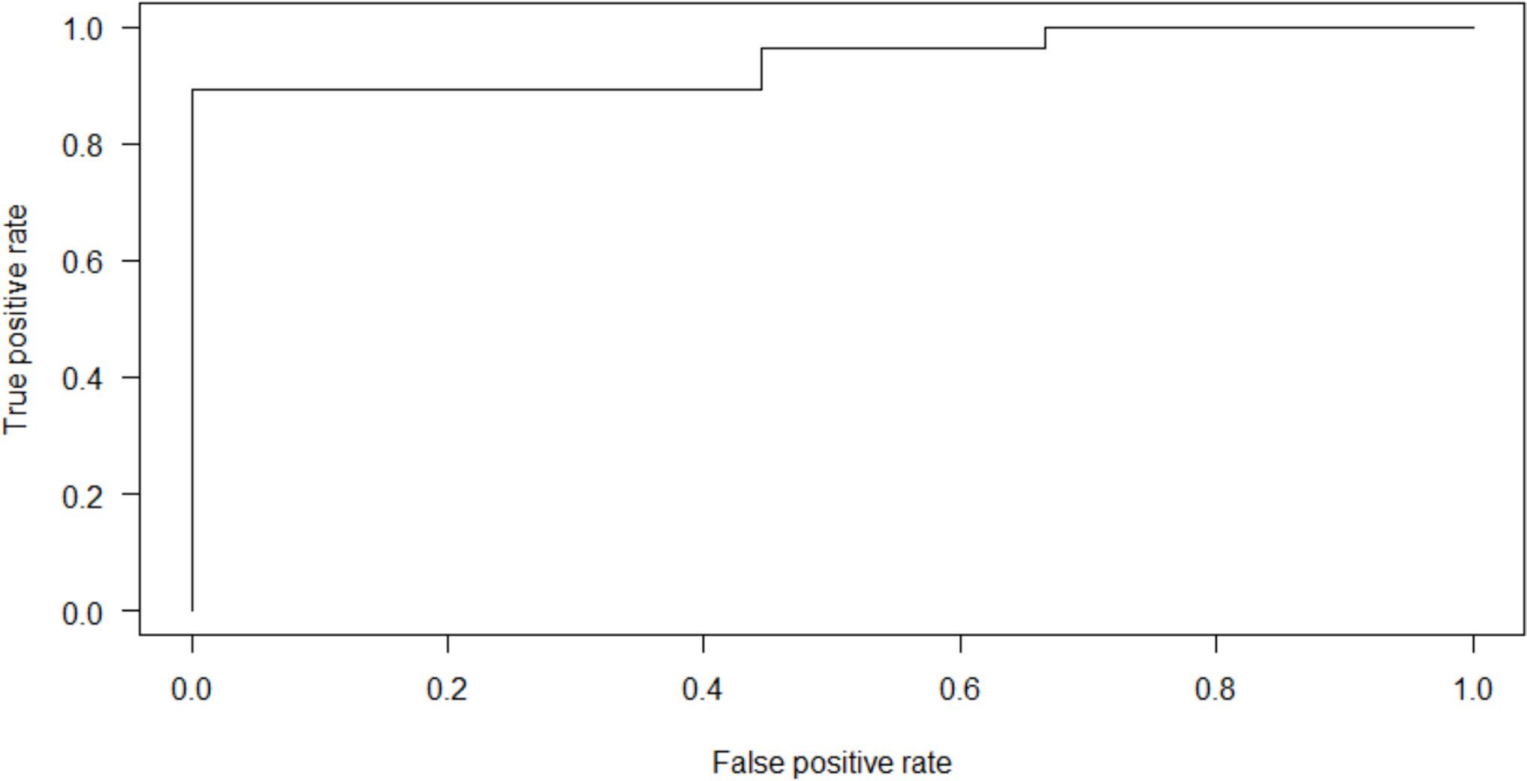
Receiver operator curve displaying false positive rate by true positive rate

ROC curves were generated using the *ROCR* package. Figure 1 displays the receiver operator curve displaying the false positive rate by the true positive rate. In this study, “positive” refers to an instance for which the model predicts success in treatment, and “negative” refers to a prediction of unsuccessful treatment. Thus, the false positive rate is the rate at which a model incorrectly predicts success when treatment was unsuccessful, and true positive rate describes how often a model correctly predicts success among cases where success was achieved. It is notable that logistic regression models provide probability predictions on a continuum between 0 and 1, and the ROC curve thus summarizes the false positive and true positive rates corresponding to each possible decision threshold for making positive and negative predictions for the model and data under use. Area under the curve (AUC) was 0.9107, suggesting an excellent fit of the model to the data. Leave-one-out cross validation was conducted to verify these results. Our leave-one-out cross validation amounts to 38 analyses that include 37 participants each, using the held-out participant for out-of-sample prediction. We note that one model failed to converge and was removed from the cross-validation analysis, resulting in 37 analyses for cross validation. Figure 2 displays the receiver operator curve for cross validation. The resulting AUC was 0.9444, validating the results of the ROC analysis.

**Fig. 3:**
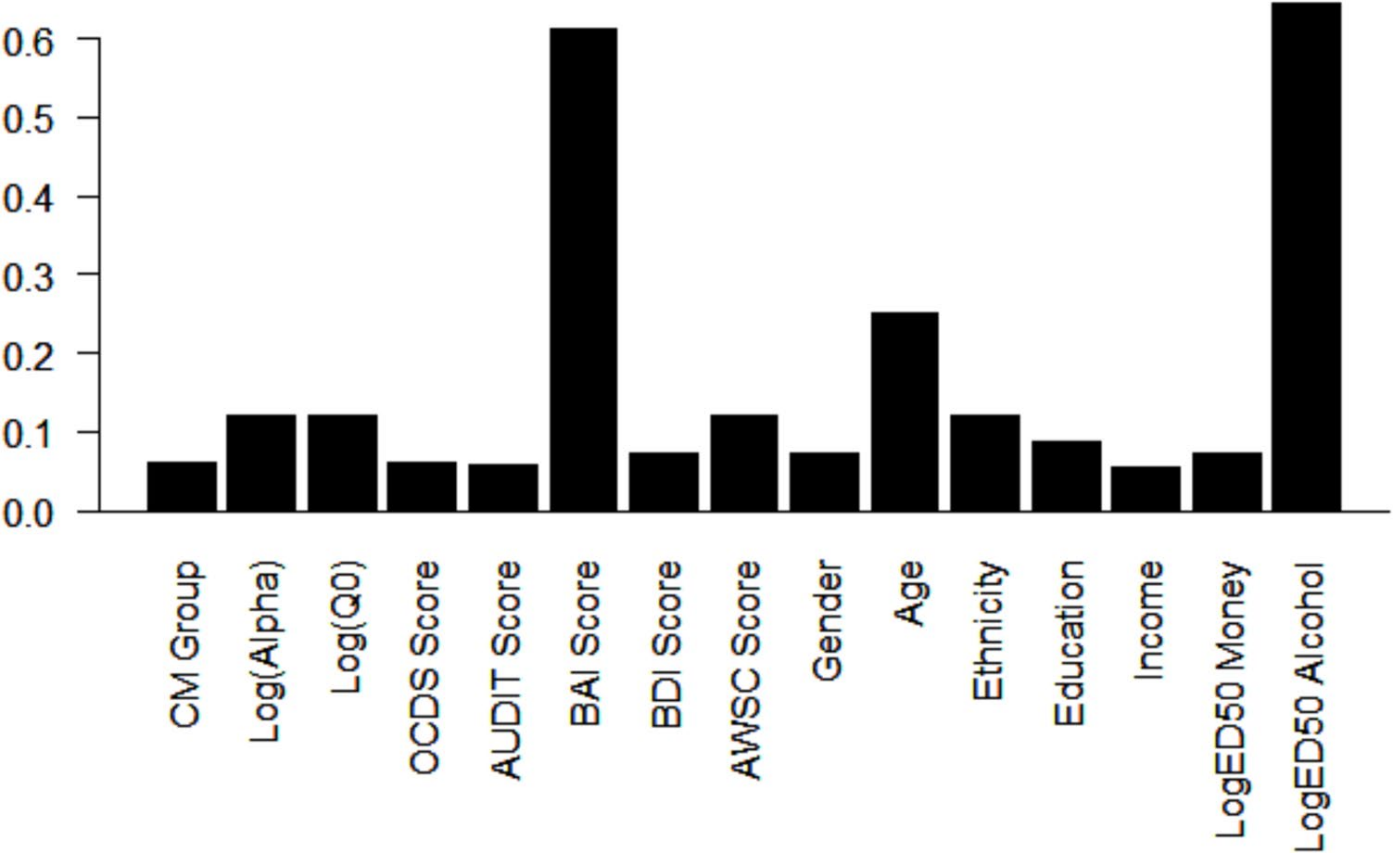
Posterior probability that each candidate predictor is non-null

Figure 3 displays posterior inclusion probabilities for each candidate predictor. These probabilities are computed using all 32,767 competing models under consideration, and may be interpreted as the probability that a given predictor is nonnull across all models (Hoeting et al., 1999). Posterior inclusion probability was greater than 0.6 for BAI score and log_10_(ED50) for alcohol. The probability of including age was greater than 0.2. All other candidate predictor probabilities were between 0 and 0.2.

## Discussion

This proof-of-concept analysis successfully yielded a model predictive of contingency management treatment outcomes in the current study sample of participants with AUD. The results of this study suggest that a model including the delay discounting parameter, log_10_(ED50) of alcohol, participant age, and BAI score may be predictive of treatment outcomes in contingency management for alcohol use. Although these results are preliminary, log_10_(ED50) for alcohol and BAI score were identified as predictors of treatment outcomes in the five top models resulting from the ROC analysis, suggesting that these variables may be important to include in future predictive models. Future research should include these variables to continue exploring their utility for use in a risk calculator that predicts treatment response to CM.

The relationship between delay discounting and treatment outcomes has been demonstrated in prior research (for a review, see Exum et al., 2023). However, findings are mixed. The variability in findings may be attributable to differences in methodology (e.g., specificity and resolution of the task; adjusting vs. fixed choices), the commodity assessed (e.g., drug, money), implementation of real or hypothetical tasks, and the discounting parameters used to predict treatment success (e.g., *k*, log(*k*); Exum et al., 2023). The studies included in the current analysis used adjusting, hypothetical delay discounting tasks. Both monetary and alcohol delay discounting were assessed. The log_10_(ED50) parameter was included in the analysis for benefits like minimizing ceiling or floor effects that may be observed with other parameters. In addition, ED50 is an easy to interpret value, as it is expressed as a unit of time. If the goal is building a risk calculator for use as a metric of individual risk factors in treatment, then using the ED50 parameter is appealing because it is easy to understand by both researchers and clinicians. Because ED50 is the inverse of *k*, it is easy to calculate from existing datasets.

Statistical model building is often nuanced. The current analysis demonstrates one potentially effective combination that may be predictive of contingency management outcomes. However, further research is needed to identify the ideal methods and parameters to include in a predictive model using a larger sample size to build a reliable risk calculator. For example, although our top model has high accuracy in predicting outcomes for the study data as evidence by high AUC, we note that the posterior model probability for this model (among the 32,767 under consideration) was only 3.9%, with the second and third best models having 3.1% and 1.7% posterior probability, respectively. These runner-up models are not bad and it seems possible that additional data collection and analysis could further refine modeling results. Based on our experience analyzing accuracy in logistic regression models, we were surprised at the very high AUC values in this pilot study for both in-sample and out-of-sample cross validation. We anticipate the possibility AUC will not be as high in other studies. Still, we recommend that future research include a measure of drug discounting in addition to monetary discounting. As shown in the current study, drug discounting may be an important and predictive decision-making process to consider. In addition, we considered treatment success as a binary outcome based on whether a threshold of 80% of sessions with low BAL was achieved. Another approach would be to create treatment effectiveness categories and use ordinal models to predict success in terms of those categories, or even to model BAL directly.

Prior research has demonstrated a comorbidity of anxiety and AUD, and anxiety is predictive of relapse posttreatment (Bradizza et al., 2006; Kushner et al., 2005). It should be noted that the current data show the results from CM treatment, but not of alcohol use after the CM condition was removed. Future research is needed to (1) verify the inclusion of BAI score in the predictive model, and (2) evaluate whether BAI score is predictive of treatment relapse post-CM treatment. It is interesting that higher BAI scores were observed in the participants who achieved treatment success. However, it should be noted that the average BAI score for the success group was 9.11, indicating mild anxiety, whereas the nonsuccess group scored 4.1, indicating minimal anxiety levels. It is possible that a small amount of anxiety may be beneficial to adhering to contingency management for alcohol use. However, these findings should be considered carefully, because they are in contrast to findings that suggests that subclinical (e.g., Crum et al., 2013) and clinical (e.g., Rabinowitz et al., 2023) anxiety are predictive of poorer treatment outcomes for AUD. Further research is needed to explore this claim.

Previous research has demonstrated a relationship between age and relapse in individuals with AUD; however, findings are mixed (for a review, see Sliedrecht et al., 2019). Age was identified as a predictive factor in the first top model in the current analysis, with younger age associated with poorer outcomes. Age did not appear as a predictor in the next four models. Ages of participants who did and did not achieve success in CM spanned across similar ranges. Participants who achieved success were aged 21–64 (*M* = 42.3), whereas participants who did not were aged 23–62 (*M* = 39.6). Because the sample size was small, it is possible that a few participants in the group that did not achieve success were clustered around the same age. This may have led to a detected difference in groups that would otherwise be washed out with a larger sample size. A larger sample size is needed in replications of this analysis.

Prior research has primarily focused on complete abstinence as a measure of treatment success. As harm reduction outcomes (e.g., reduced use) become increasingly acceptable, it may be important to redefine “treatment success.” In the current analysis, we defined treatment success as achieving 80% or more days with a BAL of less than.02. Therefore, the current results are predictive of reduced use of alcohol, rather than complete abstinence. Replications of this work should consider the inclusion of nonabstinence outcomes as potential treatment endpoints. From a therapeutic perspective, it may be important to consider alternative treatment goals to total abstinence.

This study piloted early steps for building a risk calculator predictive of the optimal incentive size to prescribe in contingency management interventions based on participant behavioral patterns and characteristics. This study provides the foundation for a body of work aimed at identifying the minimum procedural requirements needed to ensure optimal success rates in contingency management. By identifying the factors that influence the likelihood of successful contingency management, we will be able to recommend a minimum incentive amount based on scientific evidence. For example, if it is determined that low anxiety and steeper alcohol discounting predict that a participant will not be successful in contingency management, a larger incentive amount may be necessary to motivate them. The risk calculator will be used to identify the boundaries in which most participants will achieve success in contingency management. Next steps in the analysis include implementing these methods with a larger sample size, followed by developing the risk calculator. It is important to note that incentive size is only one of many manipulable aspects of CM. Future research can continue to build on this line of work by examining the relationship between participant factors and optimal incentive types, schedules of reinforcement, target behaviors, methods of measuring target behaviors, and treatment modalities.

Although the results of this analysis represent a promising framework and first step toward building a risk calculator predictive of treatment outcomes, this study was limited in that it included a small sample size. A majority of the participants in this sample were able to achieve success (i.e., 80% abstinence) under contingency management conditions (*n* = 28). Only 26.3% of participants did *not* achieve 80% abstinence. Because fewer participants were included in the nonabstinence group, each data point had potential to affect the average more heavily than in the nonabstinence group. Future research should include a sample size powered to identify between-group differences.

A second limitation is that this study focused primarily on alcohol use. Additional research is needed to determine whether the factors predictive of treatment outcomes in this study would generalize to other substance use. Future research should include the variables identified in this study to assess the replicability of these findings. In particular, it is important that future research includes delay discounting of drugs, not just money. Current research scarcely includes delay discounting of substances in analyses. However, the results of this study suggest that delay discounting of drugs may be an important predictive factor. Third, it should be noted that this study included two types of CM interventions. Deposit contracts CM is fundamentally a negative reinforcement procedure when target behavior is observed, wherein the aversive condition (i.e., unavailability of deposited money) is lessened by engaging in the desired behavior (i.e., reduced alcohol use). Incentive-based CM, on the other hand, is fundamentally a positive reinforcement procedure when an increase in target behavior is observed, which can be attributed to the addition of a stimulus (i.e., money) that improves the environmental condition. In addition to different controlling variables, there is potential for selection bias in deposit contracts CM, as it requires a lump sum of money deposited and earned back. Thus, it may necessarily include participants with a higher SES. That said, in the present analysis, income variables did not differ significantly between CM groups. CM Group was included as a candidate variable. It was not predictive of success in CM within the top 17 models, providing further confidence in the results of this analysis.

Finally, this study was limited in that it included a relatively homogenous sample. Participants were primarily white and non-Hispanic. Therefore, the results of this analysis may not generalize to non-white individuals. In addition, participants were only included if they did not meet *DSM-V* criteria for substances other than alcohol, nicotine, or caffeine. Because substance use disorders are often comorbid, future research should allow for the inclusion of people who meet *DSM-V* criteria for other substances. Inclusion of other substance use disorders will improve external validity of this tool.

Identifying a predictive model of success in CM is an important first step toward building a risk calculator. The results of this analysis demonstrate the utility of the ROC analysis as a method for identifying a predictive model. Further research is needed to replicate and verify the findings of the current analysis. As CM continues moving from research to practice, a predictive model of success will become increasingly important to ensure that individuals at higher risk receive tailored treatment that improves the likelihood they will achieve success in treatment.

## Data Availability

Data and code are available in the supplemental materials.

## Footnotes

1 The scaling constant is typically denoted as *k*, but was changed to *s* for the current analysis to avoid confusion with the discount rate *k.*

## References

[CR1] Anton, R. F., Moak, D. H., & Latham, P. (1995). The obsessive compulsive drinking scale: A self-rated instrument for the quantification of thoughts about alcohol and drinking behavior. *Alcoholism, Clinical and Experimental Research,**19*(1), 92–99. 10.1111/j.1530-0277.1995.tb01475.x 7771669 10.1111/j.1530-0277.1995.tb01475.x

[CR2] Babor, T. F., Higgins-Biddle, J. C., Saunders, J. C., & Monteiro, M. G. (2001). *The Alcohol Use Disorders Identification Test: Guidelines for use in primary care* (2nd ed). World Health Organization.

[CR3] Beck, A. T., Epstein, N., Brown, G., & Steer, R. A. (1988). An inventory for measuring clinical anxiety: Psychometric properties. *Journal of Consulting and Clinical Psychology,**56*, 893–897. 10.1037/0022-006x.56.6.893 3204199 10.1037//0022-006x.56.6.893

[CR4] Beck, A. T., Ward, C. H., Mendelson, M., Mock, J., & Erbaugh, J. (1961). An inventory for measuring depression. *Archives of General Psychiatry,**4*, 561–571. 10.1001/archpsyc.1961.01710120031004 13688369 10.1001/archpsyc.1961.01710120031004

[CR5] Bickel, W. K., Johnson, M. W., Koffarnus, M. N., MacKillop, J., & Murphy, J. G. (2014). The behavioral economics of substance use disorders: Reinforcement pathologies and their repair. *Annual Review of Clinical Psychology,**10*, 641–677. 10.1146/annurev-clinpsy-032813-153724 24679180 10.1146/annurev-clinpsy-032813-153724 PMC4501268

[CR6] Biswal, B., Bora, S., Anand, R., Bhatia, U., Fernandes, A., Joshi, M., & Nadkarni, A. (2024). A systematic review of interventions to enhance initiation of and adherence to treatment for alcohol use disorders. *Drug and Alcohol Dependence,**263*, 112429. 10.1016/j.drugalcdep.2024.112429 39232484 10.1016/j.drugalcdep.2024.112429

[CR7] Bradizza, C. M., Stasiewicz, P. R., & Paas, N. D. (2006). Relapse to alcohol and drug use among individuals diagnosed with co-occurring mental health and substance use disorders: A review. *Clinical Psychology Review,**26*(2), 162–178. 10.1016/j.cpr.2005.11.005 16406196 10.1016/j.cpr.2005.11.005

[CR8] Coughlin, L. N., Salino, S., Jennings, C., Lacek, M., Townsend, W., Koffarnus, M. N., & Bonar, E. E. (2023). A systematic review of remotely delivered contingency management treatment for substance use. *Journal of Substance Use & Addiction Treatment,**147*, 208977. 10.1016/j.josat.2023.208977 36804352 10.1016/j.josat.2023.208977 PMC10936237

[CR9] Crum, R. M., La Flair, L., Storr, C. L., Green, K. M., Stuart, E. A., Alvanzo, A. A. H., Lazareck, S., Bolton, J. M., Robinson, J., Sareen, J., & Mojtabai, R. (2013). Reports of drinking to self-medicate anxiety symptoms: Longitudinal assessment for subgroups of individuals with alcohol dependence. *Depression and Anxiety,**30*(2), 174–183. 10.1002/da.22024 23280888 10.1002/da.22024 PMC4154590

[CR10] De Crescenzo, F., Ciabattini, M., D’Alò, G. L., De Giorgi, R., Del Giovane, C., Cassar, C., et al. (2018). Comparative efficacy and acceptability of psychosocial interventions for individuals with cocaine and amphetamine addiction: A systematic review and network meta-analysis. *PLoS Medicine,**15*(12), Article e1002715. 10.1371/journal.pmed.1002715 30586362 10.1371/journal.pmed.1002715 PMC6306153

[CR11] Dutra, L., Stathopoulou, G., Basden, S. L., Leyro, T. M., Powers, M. B., & Otto, M. W. (2008). A meta-analytic review of psychosocial interventions for substance use disorders. *American Journal of Psychiatry,**165*(2), 179–187. 10.1176/appi.ajp.2007.06111851 18198270 10.1176/appi.ajp.2007.06111851

[CR12] Exum, A. C., Sutton, C. A., Bellitti, J. S., Yi, R., & Fazzino, T. L. (2023). Delay discounting and substance use treatment outcomes: A systematic review focused on treatment outcomes and discounting methodology. *Journal of Substance Use & Addiction Treatment,**149*, 209037. 10.1016/j.josat.2023.209037 37072099 10.1016/j.josat.2023.209037 PMC10429418

[CR13] Foster, S. E., DePhilippis, D., & Forman, S. (2019). I’s” on the prize: A systematic review of individual differences in Contingency Management treatment response. *Journal of Substance Abuse Treatment,**100*, 64–83. 10.1016/j.jsat.2019.03.001 30898330 10.1016/j.jsat.2019.03.001 PMC7678142

[CR14] Ginley, M. K., Pfund, R. A., Rash, C. J., & Zajac, K. (2021). Long-term efficacy of contingency management treatment based on objective indicators of abstinence from illicit substance use up to 1 year following treatment: A meta-analysis. *Journal of Consulting & Clinical Psychology,**89*(1), 58–71. 10.1037/ccp0000552 33507776 10.1037/ccp0000552 PMC8034391

[CR15] Hoeting, J. A., Madigan, D., Raftery, A. E., & Volinsky, C. T. (1999). Bayesian model averaging: A tutorial. *Statistical Science,**14*(4), 382–401.

[CR16] Kass, R. E., & Raftery, A. E. (1995). Bayes factors. *Journal of the Statistical Association,**90*(430), 773–795. 10.1080/0161459.1995.10476572

[CR17] Kaufman, V., Tomlinson, D. C., Hellman, L., Lin, L. A., Fernandez, A. C., & Coughlin, L. N. (2025). The path forward for substance use disorder treatment using contingency management under sect. 1115 demonstration waivers. *Substance Abuse Treatment, Prevention, and Policy,**20*(1), Article 37. 10.1186/s13011-025-00666-6 41029709 10.1186/s13011-025-00666-6 PMC12486863

[CR18] Koffarnus, M. N., & Bickel, W. K. (2014). A 5-trial adjusting delay discounting task: Accurate discount rates in less than one minute. *Experimental and Clinical Psychopharmacology,**22*(3), 222–228. 10.1037/a0035973 24708144 10.1037/a0035973 PMC4461028

[CR19] Koffarnus, M. N., Bickel, W. K., & Kablinger, A. S. (2018). Remote alcohol monitoring to facilitate incentive-based treatment for alcohol use disorder: A randomized trial. *Alcoholism, Clinical and Experimental Research,**42*(12), 2423–2431. 10.1111/acer.13891 30335205 10.1111/acer.13891 PMC6286218

[CR20] Koffarnus, M. N., Franck, C. T., Stein, J. S., & Bickel, W. K. (2015). A modified exponential behavioral economic demand model to better describe consumption data. *Experimental and Clinical Psychopharmacology,**23*(6), 504–512. 10.1037/pha0000045 26280591 10.1037/pha0000045 PMC4854291

[CR21] Koffarnus, M. N., Kablinger, A. S., Kaplan, B. A., & Crill, E. M. (2021). Remotely administered incentive-based treatment for alcohol use disorder with participant-funded incentives is effective but less accessible to low-income participants. *Experimental and Clinical Psychopharmacology,**29*(5), 555–565. 10.1037/pha0000503 34110885 10.1037/pha0000503 PMC8943847

[CR22] Kushner, M. G., Abrams, K., Thuras, P., Hanson, K. L., Brekke, M., & Sletten, S. (2005). Follow-up study of anxiety disorder and alcohol dependence in comorbid alcoholism treatment patients. *Alcoholism, Clinical and Experimental Research,**29*(8), 1432–1443. 10.1097/01.alc.0000175072.17623.f8 16131851 10.1097/01.alc.0000175072.17623.f8

[CR23] MacKillop, J., Amlung, M. T., Few, L. R., Ray, L. A., Sweet, L. H., & Munafò, M. R. (2011). Delayed reward discounting and addictive behavior: A meta-analysis. *Psychopharmacology,**216*, 305–321. 10.1007/s00213-011-2229-0 21373791 10.1007/s00213-011-2229-0 PMC3201846

[CR24] Mazur, J. E. (1987). An adjusting procedure for studying delayed reinforcement. In M. L. Commons, J. E. Mazur, J. A. Nevin, & H. Rachlin (Eds.), *The effect of delay and of intervening events on reinforcement value* (pp. 55–73). Lawrence Erlbaum Associates.

[CR25] McLeod, A. I., Xu, C., & Lai, Y. (2022). *Best subset GLM and regression utilities (0.37.3)*. [Computer software]. https://cran.r-project.org/web/packages/bestglm/bestglm.pdf

[CR26] Minhas, M., Oshri, A., Amlung, M., Dennhardt, A., Ferro, M., Halladay, J., Munn, C., Tucker, J., Murphy, J., & MacKillop, J. (2020). Latent profile analysis of heavy episodic drinking in emerging adults: A reinforcer pathology approach. *Alcoholism, Clinical and Experimental Research,**44*(10), 2130–2140. 10.1111/acer.14438 32965723 10.1111/acer.14438 PMC7841846

[CR27] Petry, N. M. (2001). Delay discounting of money and alcohol in actively using alcoholics, currently abstinent alcoholics, and controls. *Psychopharmacology,**154*(3), 243–250. 10.1007/s002130000638 11351931 10.1007/s002130000638

[CR28] Pittman, B., Gueorguieva, R., Krupitsky, E., Rudenko, A. A., Flannery, B. A., & Krystal, J. H. (2007). Multidimensionality of the alcohol withdrawal symptom checklist: A factor analysis of the Alcohol Withdrawal Symptom Checklist and CIWA-Ar. *Alcoholism, Clinical and Experimental Research,**31*(4), 612–618. 10.1111/j.1530-0277.2007.00345.x 17374040 10.1111/j.1530-0277.2007.00345.x

[CR29] Rabinowitz, J. A., Ellis, J. D., Wells, J., Strickland, J. C., Maher, B. S., Hobelmann, J. G., & Huhn, A. (2023). Correlates and consequences of anxiety and depressive symptom trajectories during early treatment for alcohol use. *Alcohol,**108*, 44–54. 10.1016/j.alcohol.2022.11.005 36473635 10.1016/j.alcohol.2022.11.005 PMC10033438

[CR30] Robin, X., Turck, N., Hainard, A., Tiberti, N., Lisacek, F., Sanches, J., Müller, M., Siegert, S., Doering, M., & Billings, Z. (2023). *Display and analyze ROC curves (1.18.4).* [Computer software]. https://cran.r-project.org/web/packages/pROC/pROC.pdf

[CR31] Rowlett, J. K. (2000). A labor-supply analysis of cocaine self-administration under progressive-ratio schedules: Antecedents, methodologies, and perspectives. *Psychopharmacology,**153*(1), 1–16. 10.1007/s002130000610 11255919 10.1007/s002130000610

[CR32] Rzeszutek, M. J., Gipson-Reichardt, C. D., Kaplan, B. A., Koffarnus, M. N. (2022). Using crowdsourcing to study the differential effects of cross-drug withdrawal for cigarettes and opioids in a behavioral economic demand framework. *Experimental and Clinical Psychopharmacology, 30*(4), 453-465. 10.1037/pha0000558 10.1037/pha0000558 PMC9308700 35201826

[CR33] Sing, T., Sander, O., Beerenwinkel, N., Lengauer, T., Unterthiner, T., & Ernset., F. G. M. (2020). *ROCR: Visualizing the performance of scoring classifiers.* [Computer software]. https://cran.r-project.org/web/packages/ROCR/index.html

[CR34] Sliedrecht, W., de Waart, R., Witkiewitz, K., & Roozen, H. G. (2019). Alcohol use disorder relapse factors: A systematic review. *Psychiatry Research,**278*, 97–115. 10.1016/j.psychres.2019.05.038 31174033 10.1016/j.psychres.2019.05.038

[CR35] Traxler, H. K., Kaplan, B. A., Rzeszutek, M. R., Franck, C. T., & Koffarnus, M. N. (2023). Interest in and perceived effectiveness of contingency management among alcohol drinkers using behavioral economic purchase tasks. *Experimental and Clinical Psychopharmacology,**31*(1), 127–139. 10.1037/pha0000580 35708948 10.1037/pha0000580 PMC10103538

[CR36] Tucker, J. A., Cheong, J., Chandler, S. D., Lambert, B. H., Kwok, H., & Pietrzak, B. (2016). Behavioral economic indicators of drinking problem severity and initial outcomes among problem drinkers attempting natural recovery: A cross-sectional naturalistic study. *Addiction,**111*(11), 1956–1965. 10.1111/add.13492 27318078 10.1111/add.13492 PMC5056809

[CR37] White House. (2021, April 1). *Biden-Harris administration announces first-year drug policy priorities* [Press release]. https://www.whitehouse.gov/ondcp/briefing-room/2021/04/01/biden-harris-administration-announces-first-year-drug-policy-priorities/

[CR38] Wickham, H. (2023). Tidyverse: Easily install and load the “tidyverse.” [Computer software]. https://cran.r-project.org/package=tidyverse

[CR39] Yoon, J. H., & Higgins, S. T. (2008). Turning k on its head: Comments on use of an ED50 in delay discounting research. *Drug and Alcohol Dependence,**95*(1–2), 169–172. 10.1016/j.drugalcdep.2007.12.011 18243583 10.1016/j.drugalcdep.2007.12.011 PMC2435271

[CR40] Zvorsky, I., Nighbor, T. D., Kurti, A. N., DeSarno, M., Naude, G., Reed, D. D., & Higgins, S. T. (2019). Sensitivity of hypothetical purchase task indices when studying substance use: A systematic literature review. *Preventive Medicine,**128*, Article 105789. 10.1016/j.ypmed.2019.105789 31400376 10.1016/j.ypmed.2019.105789 PMC6879840

